# Increasing densities of Pacific crown-of-thorns starfish (*Acanthaster* cf. *solaris*) at Lizard Island, northern Great Barrier Reef, resolved using a novel survey method

**DOI:** 10.1038/s41598-023-46749-x

**Published:** 2023-11-07

**Authors:** Josie F. Chandler, Deborah Burn, Ciemon F. Caballes, Peter C. Doll, Sarah L. T. Kwong, Bethan J. Lang, Kai I. Pacey, Morgan S. Pratchett

**Affiliations:** 1 College of Science and Engineering, James Cook University, Townsville, QLD 4811 Australia; 2 Marine Laboratory, University of Guam, Mangilao, Guam, 96923 USA; 3 Australian Institute of Marine Science, PMB 3, Townsville, QLD 4810 Australia

**Keywords:** Marine biology, Population dynamics

## Abstract

Recurrent population irruptions of Pacific crown-of-thorns starfish (CoTS, *Acanthaster* cf. *solaris*) are among the foremost causes of coral mortality on Australia’s Great Barrier Reef (GBR). Early intervention during the initiation of new population irruptions represents the best opportunity to effectively manage this threat. However, current survey methods are not sufficiently sensitive to detect changes in CoTS densities during the early onset of population irruptions. Using scooter-assisted large area diver-based (SALAD) surveys, this study revealed increasing densities of CoTS at Lizard Island from 2019 to 2022. Inferred densities of adult CoTS (which account for distinct sets of observed feeding scars where starfish were not detected) increased from 4.90 ha^−1^ (± 0.85 SE) in 2019 to 17.71 ha^−1^ (± 2.3 SE) in 2022. A wide range of size classes were recorded suggesting that recruitment over several years is contributing to increasing densities. Importantly, the sustained density increases reported here denote that renewed CoTS population irruptions may soon become fully established at Lizard Island and more broadly in the northern GBR, especially without early intervention through effective population management.

## Introduction

Population irruptions of crown-of-thorns starfish (CoTS, *Acanthaster* spp.) are one of the foremost causes of coral mortality throughout Indo-Pacific coral reefs^1–5^, contributing to widespread declines in coral cover and diversity^6–9^. The initiation of population irruptions (often referred to as ‘primary outbreaks’; sensu Moran^6^, Endean^10^) is increasingly attributed to progressive build-up of CoTS densities due to successive recruitment events and cumulative increases in their local reproductive output (e.g., Pratchett^11^, Wooldridge and Brodie^12^). The underlying mechanism(s) that cause or facilitate population irruptions are largely unresolved^13,14^, though there are persistent theories ascribing some role for anthropogenic activities. More specifically, over-fishing of key predatory species, such as giant triton *Charonia tritonis*^15^ and predatory fishes^16–18^ may release CoTS from predator-mediated population regulation, and thereby facilitate increased rates of recruitment and adult population growth (*Predator Removal Hypothesis*^15^). This hypothesis has been corroborated by evidence of increased incidence and severity of population irruptions in areas subject to sustained fishing pressure^19–21^. Alternatively, the *Terrestrial Runoff Hypothesis*^22^ proposes that pulses of increased nutrients (and hence larval food availability) caused by heavy rainfall on high islands may have released planktotrophic CoTS larvae from food limitation, elevating their developmental success and survival rates and therefore exacerbating population growth^22–24^. However, it is still unknown whether low nutrient conditions would sufficiently constrain larval survival to prevent the initiation of population irruptions^25,26^.

Debate surrounding the putative cause(s) of population irruptions remains ongoing, with no single hypothesis gaining unequivocal support^13^. It is also possible that spatiotemporal variation in CoTS abundance is attributable to inherent life-history traits of CoTS (e.g., extreme fecundity^13,27–29^). Local hydrodynamic patterns have been proposed as a potential mechanism for larval retention, leading to higher-than-normal recruitment success at specific reef locations^30,31^ and could explain why some reefs or regions are more predisposed to self-seeding^24,32^. Regardless of the proximate cause(s) of population irruptions, population irruptions (or ‘primary outbreaks’) build up over time and give rise to extremely large quantities of planktonic larvae which are thought to spread to nearby and downstream reefs, resulting in subsequent population irruptions termed ‘secondary outbreaks’ (sensu Endean^10^). Effective management is conditional upon improved understanding of the causes(s) of population irruptions, particularly pertaining to the potential role of anthropogenic drivers.

Early detection (if not prevention) of increasing starfish densities represents the best opportunity to prevent the initiation and spread of population irruptions and mitigate their destructive impact on coral assemblages^33,34^. However, our ability to detect the onset of population irruptions has been severely constrained by difficulties in surveying CoTS outside of peak adult densities^33,35^. This is partly due to the attention-deficit cycle of CoTS research and funding that closely mirrors the cycles of CoTS population irruptions^36^, though there are also substantial logistical constraints to effectively monitoring CoTS, especially low-density populations. Current surveillance is largely reliant on manta tows^37,38^, which provide only very coarse estimates of CoTS densities^35,39^. Such methods have utility in establishing where population irruptions presently exist and have been vital in directly informing the Crown-of-thorns Starfish Control Program on Australia’s Great Barrier Reef (GBR)^40^. However, they are not sufficiently sensitive to reliably detect adult density changes in locations where CoTS population irruptions are not yet fully established.

Our inability to detect changes in starfish densities during the early stages of CoTS population irruptions has prevented us from establishing precisely when localised and system-wide population irruptions begin, thereby hindering our understanding of their root cause(s). Population irruptions have historically been recognised once sufficiently high densities of adult CoTS have surpassed a notional abundance or density threshold (i.e., 0.22 CoTS per manta tow or 15 CoTS ha^−1^ sensu Moran and De’ath^41^; 10 CoTS ha^−1^ sensu Keesing and Lucas^42^) defined for management and research purposes. Under this definition, however, the purported timing of initiation relies on the detection of a population irruption, which likely occurs years after densities first started building, thereby masking the potential root cause(s) of initial population growth. For the earliest possible detection of population irruptions, it is thus not only absolute CoTS densities which are noteworthy, but equally the first signs and rate of population growth. Because current surveillance approaches do not have the capacity to detect these subtle changes, developing a survey method which overcomes the logistical difficulties of surveying highly cryptic, low-density CoTS would enable monitoring of population dynamics at a much higher resolution and resolve these inherent issues pertaining to the detection of population build-up.

Since the 1960s there have been four distinct waves of CoTS population irruptions documented on the GBR^11,34,43,44^, with their initiation and spread seeming to follow a relatively consistent spatiotemporal pattern. With each of these cycles, successive, elevated annual recruitment has been proposed to first occur within a finite area in the Northern GBR (14° S to 17° S)^11,32,45,46^, which has consequently been termed the ‘Initiation Box’^46–48^. Based on the periodicity (14–17 years) of past cycles, it is expected that a new wave of population irruptions will commence in the northern GBR between 2025–2027^34^. The purpose of this study was to undertake CoTS population monitoring of a key geographic area within the ‘Initiation Box’ (Lizard Island) at a higher resolution than has previously been possible. This fine-scale surveillance using a novel survey method would attempt to detect the first signs of CoTS population build-up, which would mark a key development in our early detection capability. Moreover, population size structure data collected during surveys offers important insights into historic recruitment and population dynamics in this key location, which may consequently corroborate or contradict putative hypotheses for the initiation of CoTS population irruptions.

## Methods

### Study site

**Figure 1:**
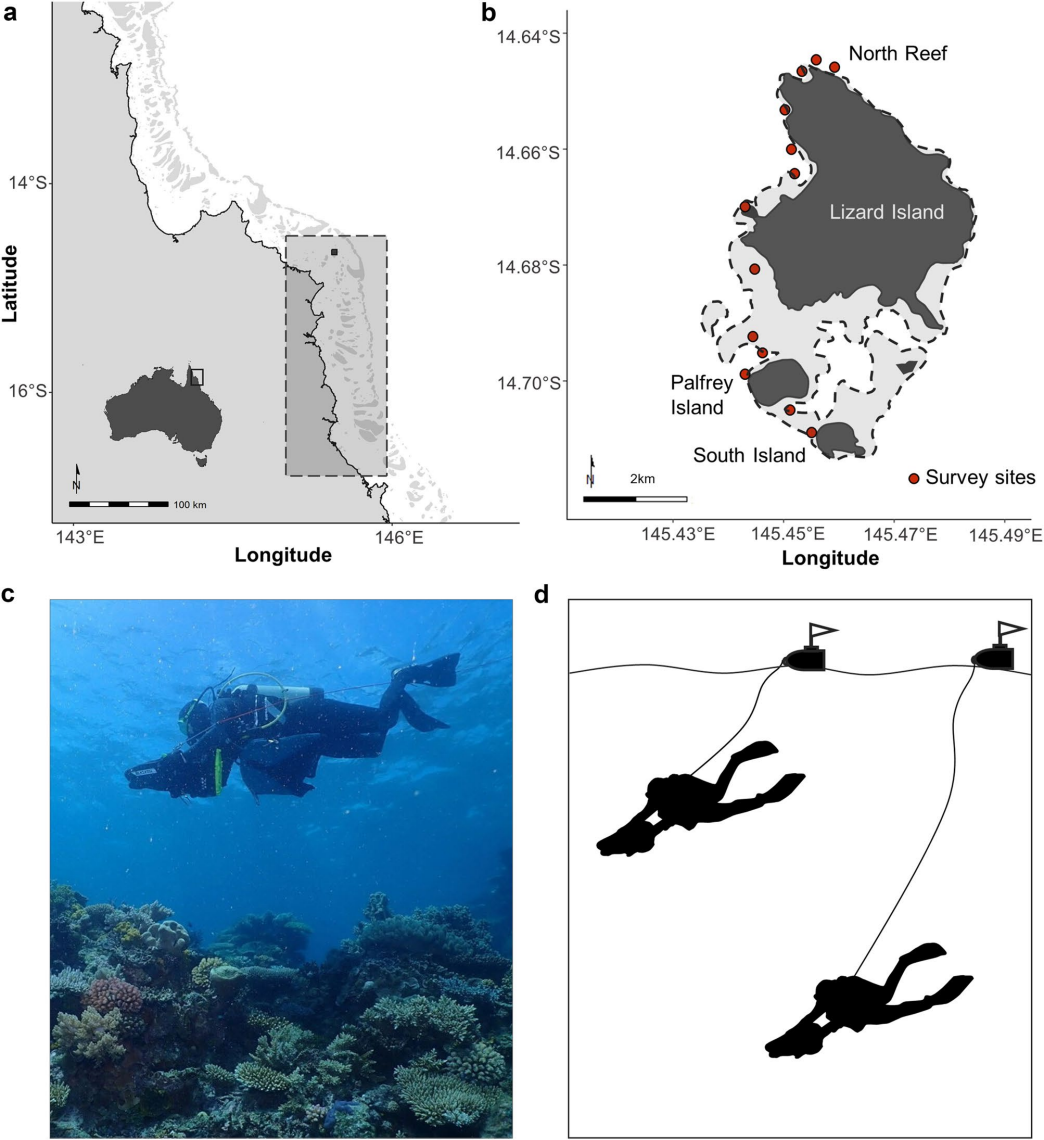
‘SALAD surveys’ conducted at Lizard Island. (**a**) Lizard Island’s location in the Northern Great Barrier Reef, also shown is the putative ‘Initiation Box’ (dashed box), (**b**) Lizard Island area with survey locations marked in red, (**c**) SALAD survey conducted with a Seascooter, (**d**) Methodology: searches are conducted at two depths simultaneously, with GPS units towed along the surface in floating housings.

This study was conducted on reefs around Lizard Island (14°40′S, 145°27′E) in the northern GBR, Australia (Fig. 1a,b). Lizard Island’s location in the northern extent of the purported ‘Initiation Box’ (Fig. 1a) has made it a key site for investigating the initiation and southward spread of CoTS population irruptions. Since monitoring of GBR reefs for CoTS began in 1966^6^, Lizard Island has reported elevated densities of CoTS that surpass indicative thresholds of a population irruption on three occasions^6,11,32^. Although there is no empirical data on the earliest stages of population build-up during each of these cycles, it is presumed that the populations increased slowly and progressively to start with, followed by a pattern of rapid and dramatic population growth and eventually a precipitous decline^11,49,50^. On each occasion, pronounced increases in population size at Lizard Island and surrounding reefs have directly preceded the southward spread of ‘secondary outbreaks’^51,52^, and this area has consequently been hypothesised to be a ‘seed area’ for region-wide population irruptions^11,44^. Understanding the specific dynamics of CoTS populations at Lizard Island could hold critical answers to how and why population irruptions begin^47^.

In this present study, a total of 13 survey sites were selected around Lizard Island, predominantly on the leeward side of the island (Fig. 1b), owing to weather constraints. The surveyed region extends from North Reef in the north, to Palfrey and South Islands in the south (Fig. 1b). Each of these sites were extensively surveyed in four consecutive years from 2019 to 2022.

### SALAD survey methods

To effectively survey CoTS at very low densities, when individuals are known to be widely dispersed^1^ and generally cryptic during daylight hours^53–55^, a novel survey method was developed, namely Scooter-Assisted Large Area Diver-based (SALAD) surveys (Fig. 1c,d). To account for the low densities of CoTS, it was necessary to survey large spatial areas, while also allowing for intensive searching for CoTS concealed within the reef matrix. Therefore, this novel survey method involved in situ CoTS surveys using Yamaha (500Li) Seascooters (Fig. 1c,d), which enabled greatly increased areal extent of individual surveys, while ensuring divers had full autonomy to vary the speed of travel during SALAD surveys, depending on habitat complexity and the presence of feeding scars, and could stop as necessary to search for CoTS. During SALAD surveys divers were able to survey up to 1.1 ha (11,000 m^2^) of reef area per dive.

SALAD surveys were undertaken by pairs of SCUBA divers, where each diver searched a distinct depth range or habitat, to the extent that was possible given the reef topography and water visibility. Each diver surveyed 2.5 m either side of their median plane, for a total of a 5-m wide survey area. Survey pairs therefore remained > 5 m apart to avoid pseudoreplication of observations (i.e., re-surveying the same area). Survey pairs were divided into two roles, specifically one diver (shallow) would survey along the reef crest (2–4 m), whilst the other diver (deep) would survey along reef slope (5–9 m); adjusting as necessary to remain within sight of each other.

In order to determine the total distance surveyed (and thereby determine overall area searched) during each SALAD survey, a surface-float housing a GPS unit (Garmin eTrex 10) was tethered to each diver (see Fig. 1d). The GPS unit recorded the start and end points of each survey track, and continuously recorded the GPS location at 60 s intervals. Recorded GPS track data was converted to CSV format and tracks were then scrubbed in statistical software R^56^ using speed, distance, and time parameters to identify and remove any track segments where divers were not engaged in conducting surveys (e.g., moving to or from site in boat). Distance was then calculated for each cleaned track using the ‘distHaversine’ function of the R package ‘geosphere’^57^. The resulting survey-specific search area per diver was specified to be 5 m wide (i.e., 2.5 m either side) along the central line of the survey path, which was assumed to represent the diver’s median plane.

Given COTS are generally cryptic, especially at small size and during daylight hours^53,55^, the initial detection of individuals is generally based on the appearance of conspicuous feeding scars, rather than direct detection of the starfish^39,58^. As such, discounting individuals which are not readily visible during daytime searches is thought to considerably underestimate CoTS abundances^35,39^. Importantly, the SALAD method allows rigorous assessment of coral colonies to distinguish CoTS feeding scars from other common stressors (e.g., coral disease or predation by *Drupella* spp.). When feeding scars are clearly discernible and easily attributed to individual starfish (which is only possible at low to moderate densities), local densities of CoTS may be inferred based on the number of distinct clusters of feeding scars. Because this study hoped to capture the subtle, early changes in population densities, we recorded distinct sets of feeding scars (where no starfish was detected) in addition to CoTS counts, this was thought to represent a more accurate indicator of adult CoTS abundance on a reef where detectability is low. A feeding cluster was identified as either a trail of progressive feeding scars or a distinct patch where multiple fresh scars were present within close proximity, separated from another cluster by an expanse of reef without any feeding scars. Estimates of CoTS abundance that take account of feeding scars are referred to herein as ‘inferred’ densities (as opposed to ‘recorded’), being the number of recorded CoTS detected summed with the number of distinct sets of CoTS feeding scars not assigned to a recorded starfish (i.e., CoTS + Scars). If there was uncertainty about whether a set of scars was related to an identified starfish or a cryptic starfish, then only the actual starfish was counted. For each starfish located, the body size (i.e., maximum diameter) was recorded by measuring the longest width of the starfish using a slate ruler or measuring tape. Although the size of starfish is known to be influenced by extrinsic factors such as diet and competition^59–61^, this size-frequency distribution data provides a coarse indication of age structure and historic recruitment to the population (sensu Stump^49^, Pratchett^11^) and hence population dynamics. Based on taxon-specific size at age relationships^47^, any < 150 mm diameter CoTS likely represents recent recruitment cohorts (≤ 2 years), while observations of a wide range of size classes denote multiple recruitment events over a prolonged period. Size data were grouped into size classes with 50 mm bin ranges and subsequently used to produce size frequency distributions for each survey year.

### Statistical analyses

To investigate temporal and spatial variation in CoTS densities at Lizard Island, a generalized linear mixed effects model (GLMM) was used to model the effects of year (2019–2022) and zone (reef crest and slope). Site was included as a random effect with random intercepts to account for spatial variability in CoTS densities. CoTS densities were modelled against a Poisson distribution with a log link function, whilst transect length was added as an offset to account for the differing areal extent of each survey. Tukey’s pairwise comparisons were calculated using estimated marginal means to test for significant differences in CoTS abundances among years. 95% confidence intervals (CI) were generated for these pairwise comparisons, whereby statistically significant differences are determined where CIs do not overlap 1. Surveys were conducted in June–August and October–December, however, data were pooled by year to test for inter-annual variation as there was found to be no influence of sampling period on inferred CoTS densities (ANOVA, F_1,127_ = 1.433, p = 0.233). Statistical analyses are presented for two alternative estimates of CoTS density; one for recorded CoTS densities and one for inferred CoTS densities. To investigate temporal variation in the body size of CoTS, a second GLMM modelled the effect of year on the diameter of CoTS, including site as a random effect. The size data were modelled against a Gaussian distribution and a glmmTMB ANOVA (Wald II) was computed to test for significant variation in mean CoTS diameter among years.

All statistical analyses were performed in the R statistical and graphical environment^56^, with models built using the *glmmTMB* package^62^. For each model, goodness of fit was assessed, and combinations of fixed and random effects were tested and retained only if they improved the fit of the model, based on the Akaike Information Criterion (AIC). Each model was validated, including checks of simulated residuals and dispersion, using the *DHARMa* package^63^. The *emmeans* package^64^ was used for pairwise comparisons of estimated marginal means. Boxplots and size-frequency distribution plots were computed using the *ggplot2* package in R^65^.

## Results

### Inter-annual variation in CoTS density

**Figure 2:**
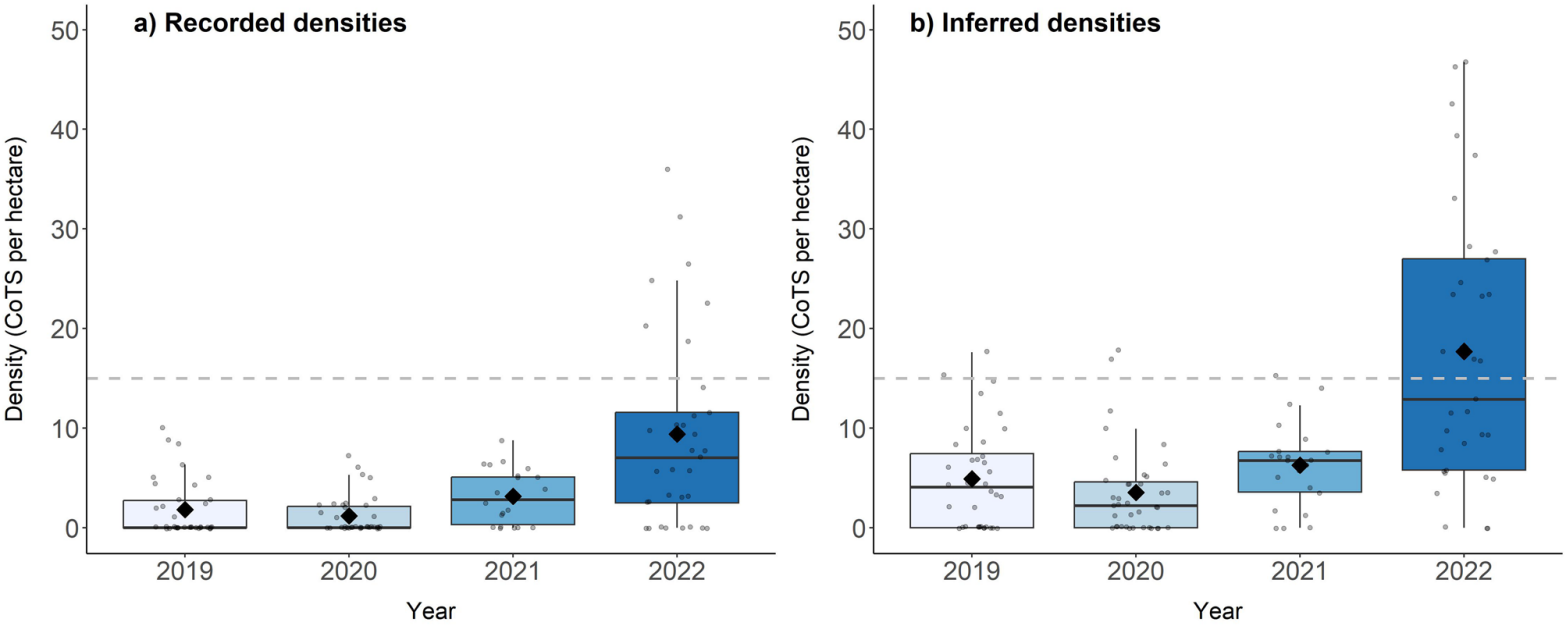
Inter-annual variation in CoTS densities at Lizard Island from 2019 to 2022. (**a**) Recorded density estimates based on starfish counts, (**b**) inferred density estimates (accounting for additional, distinct sets of feeding scars where starfish were not detected). Each grey point represents a density estimate for an individual SALAD survey. Boxes and whiskers display 25–75th and 5–95th percentile ranges, respectively. Diamonds and thick lines indicate mean and median values, respectively. Dashed line indicates the ‘outbreak threshold’ commonly used by monitoring agencies^51^.

A total of 130 SALAD surveys were conducted at Lizard Island between 2019 and 2022, encompassing a total survey area of 67.4 ha and detecting a total of 197 adult CoTS (Table 1). Over the 4 years, recorded densities of CoTS ranged from 0 to 35.9 CoTS per hectare among individual SALAD surveys, while inferred densities (including distinct sets of feeding scars) ranged from 0 to 46.8 CoTS per hectare. In 2019 and 2020, the densities of starfish remained relatively low (Fig. 2a,b), with mean recorded CoTS densities of 1.82 ha^−1^ (± 0.48 SE) in 2019 and 1.18 ha^−1^ (± 0.31 SE) in 2020 (Fig. 2a), and inferred densities of 4.90 ha^−1^ (± 0.85 SE) in 2019 and 3.53 ha^−1^ (± 0.72 SE) in 2020 (Fig. 2b).

**Table 1:** Summary of Scooter-Assisted Large Area Diver-based (SALAD) surveys conducted at Lizard Island during 2019–2022, with survey distance and survey area per survey year.

Year	No. of surveys (n)	Cumulative distance of survey paths (m)	Combined survey area (Ha)	Recorded no. of CoTS	Inferred no. of CoTS (CoTS + sets of scars)
2019	36	40,744	19.72	34	91
2020	38	42,332	21.17	26	77
2021	22	33,096	16.54	52	111
2022	34	19,999	10.00	85	161
Total	130	136,171	67.43	197	440

In 2021, mean recorded densities (3.14 ha^−1^ ± 0.58 SE) and inferred densities of CoTS (6.29 ha^−1^ ± 0.91 SE) were found to be significantly higher than in 2020 (recorded CI 1.47–4.32; inferred CI 1.26–2.51). Subsequently, surveys conducted in 2022 found further significant and considerable increases in mean CoTS densities compared to 2021 for both recorded density estimates (CI 2.37–6.76) and inferred density estimates (CI 4.36–5.47). In 2022, the mean recorded density of CoTS was 9.41 individuals ha^−1^ (± 1.66 SE), while the mean inferred density of CoTS (17.71 ha^−1^ ± 2.30 SE) exceeded the critical density threshold of 15 CoTS ha^−1^. At the survey level, CoTS densities above this density threshold were detected during 7 and 16 surveys in 2022 for recorded and inferred densities, respectively. Notably, mean densities in 2022 were substantially higher than in 2019 (recorded CI 3.62–11.78; inferred CI 2.35–4.74) and 2020 (recorded CI 5.67–17.95; inferred CI 2.88–5.53). Recorded and inferred density estimates present a similar trend of increasing densities from 2020 to 2022, whereby mean inferred density estimates were consistently higher in each of these years (Fig. 2a,b).

There was no significant effect of zone (depth) on recorded CoTS densities during any of the years (CI 0.79–1.46). Overall, the fixed effects in the model (year and zone) were found to explain 44% of variability observed in recorded (marginal R^2^ = 0.439) and inferred CoTS densities (R^2^m = 0.427), with the model explaining 70% of variation (conditional R^2^ = 0.701) for recorded density estimates and 75% for inferred density estimates (R^2^c = 0.753).

### Size structure

**Figure 3:**
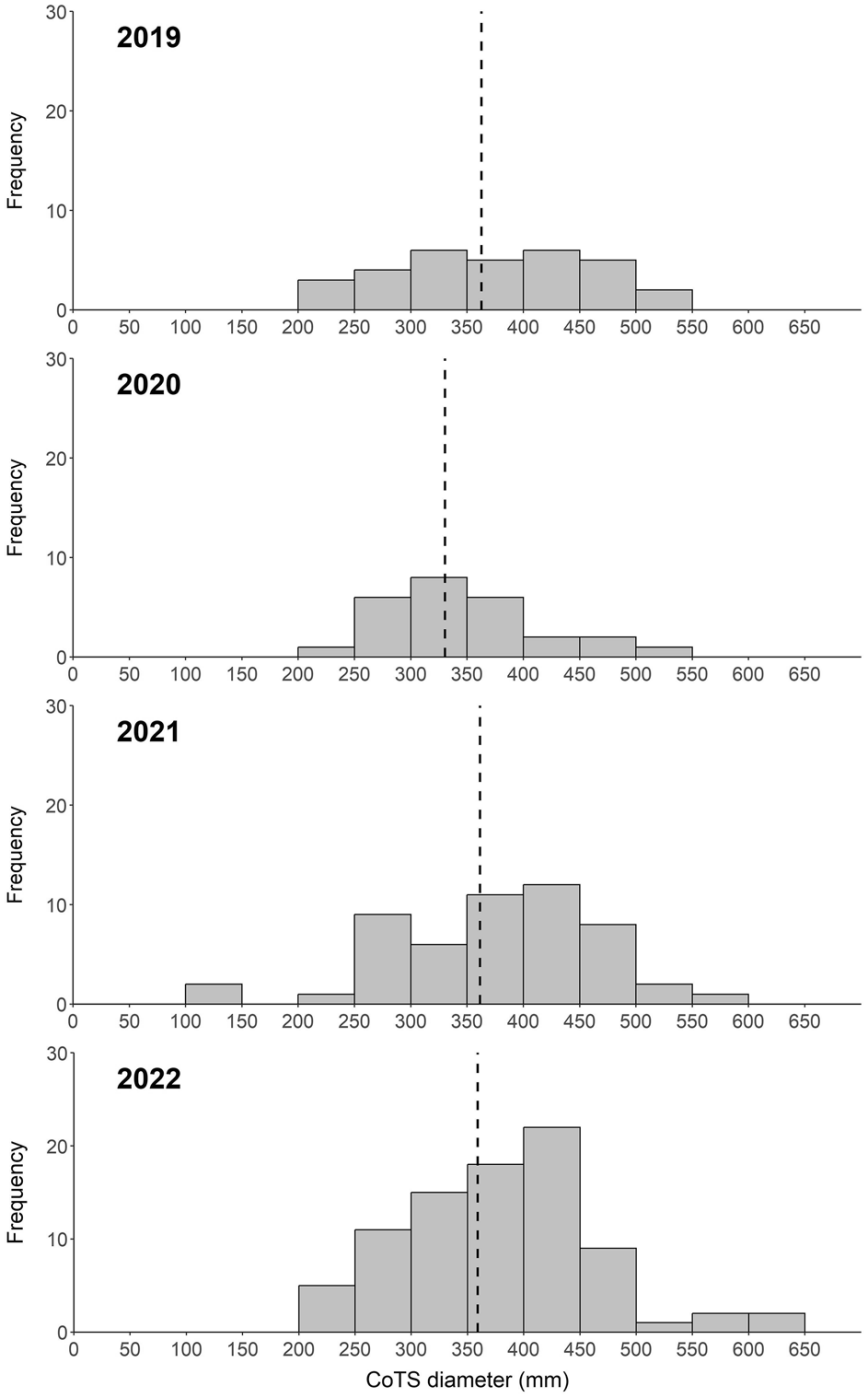
Inter-annual variation in size frequency distribution of adult CoTS recorded at Lizard Island between 2019 and 2022. Dashed line indicates the mean CoTS diameter (mm) for each survey year.

The diameter of COTS recorded during SALAD surveys ranged from 100 to 600 mm, with a broad range of sizes recorded in every year (Fig. 3). The size data GLMM reported no significant variation in mean CoTS diameter among survey years (glmmTMB ANOVA, *X*^2^ [3, n = 194] = 2.826, p = 0.419) and very few small starfish (< 150 mm diameter) were recorded (total of 3 individuals). The size-frequency distribution remained relatively consistent among years; however, slightly higher proportions of CoTS were present in the large size classes in 2021 and 2022, with some individuals between 550 to 600 mm in diameter. There was a temporal shift towards larger size classes, whereby the modal size class in 2020 was 300–350 mm, compared to 400–450 mm in years 2021 and 2022 (Fig. 3).

## Discussion

Increasing densities recorded during this study signal the onset of a renewed CoTS population irruption at Lizard Island in the northern GBR, representing the 5th documented irruption since the 1960s. Based on the historic periodicity of previous cycles, a renewed population irruption was not predicted to start until 2025–2027^34^. However, the detection (and reported initiation) of all previous CoTS population irruptions has been based solely upon the detection of absolute densities of adult CoTS above the nominal density threshold of 15 CoTS per hectare^6,11,51^. Such conspicuously high densities represent fully established population irruptions which have been identified considerably later than the initial conception and first apparent density changes would have occurred. However, there has previously been no method capable of detecting the early density changes that signify the actual onset of a population irruption, hindering both our understanding of irruption driver(s) and early intervention efforts. To resolve this long-lasting conundrum and overcome the inherent limitations of previous methods to effectively resolve low density CoTS, this study used the novel SALAD survey method, which integrates thorough CoTS searches within large area surveys. These innovative surveys reveal substantial and significant increases in CoTS densities at Lizard Island over the past few years, and inferred densities surpassed the established density threshold that signifies population irruptions in 2022. The documented increase in local densities over this multi-year period presents a strong indication that the anticipated renewed population irruption has in fact already started at this key location within the ‘Initiation Box’ and marks an important development in our capability to detect and suppress CoTS population irruptions before they take hold.

Whilst the SALAD surveys recorded significant and considerable increases in CoTS densities between 2020 and 2022, other monitoring programs operating in the region have detected no such temporal variation during the same years at Lizard Island (Emslie et al., AIMS LTMP [unpublished data]). This disparity demonstrates the value of the new SALAD survey method for reliable density estimates and, importantly, for early detection purposes. The SALAD survey method is enabling us to detect the first signs of increasing CoTS densities years before other conventional monitoring techniques. Our findings suggest that the onset of the four reported population irruptions on the GBR would have similarly been detectable years before their initiation was officially recorded, had this method been accessible at the time. The newfound capacity to monitor low-density CoTS populations has also enabled baseline density data to be collected for the first time. These data will be invaluable for improving our understanding of population dynamics and allow for future comparisons of baseline data between populations of different regions and even between different *Acanthaster* species. Most importantly, this monitoring method will allow us to detect future deviations from baseline densities, which has considerable potential as an early warning system for swift and effective management intervention.

To infer more accurate estimates of population density, this study recorded distinct sets of CoTS feeding scars, as previously implemented in the monitoring of CoTS populations^8,58^. CoTS are extremely cryptic during daytime surveys^55^, particularly at low population density, which can result in substantial density underestimation, even in the case of extensive areal searches to locate the starfish (e.g., SALAD survey method). In areas with low to intermediate CoTS densities, where the starfish are generally widely dispersed^1^, distinct sets of feeding scars can be readily attributed to a single CoTS feeding in isolation. In most such cases, the inclusion of feeding scars in density estimates will simply reduce density underestimation and provide a more accurate measure of abundance than reliance on locating the highly cryptic starfish themselves. With detectability or ‘sightability’ highlighted as a key constraint to accurate survey data for CoTS^35,39^, this inclusion of scars represents the most robust method of estimating CoTS densities by essentially buffering for any potential spatial or temporal heterogeneity in detectability which may be related to CoTS density, body size, reef complexity, season or time of day. There are, however, inherent limitations associated with this approach, particularly in areas with elevated densities, where feeding patches may overlap and sets of scars become more difficult to discern. Feeding scars may also be misassigned to CoTS which have moved on or are feeding over a large area rather than within a distinct patch^42^. On the other hand, the rigorous assessment of potential feeding scars during SALAD surveys ensures the correct differentiation from other potential stressors, such as coral disease or predation by *Drupella* spp.^66^, which contrasts another critical limitation of other survey methods to obtain accurate abundance at low CoTS densities (e.g., manta tow, remotely operated or autonomous underwater vehicles). On balance, the inclusion of feeding scars in the monitoring of CoTS populations is still more likely to underestimate than overestimate true densities. As many previous studies have not included feeding scars in published abundance estimates, the new insights gained from this data should be interpreted accordingly, especially in the context of the established threshold that signifies population irruptions.

Whilst abundance data has proven critical for capturing the initial shift from latent to proliferating population, size frequency data collected during this study provides additional insight into how this population build-up may have occurred. Inherent limitations to detecting < 100 mm CoTS hinder precise insights into recruitment patterns; however, our size frequency data allows for a coarse age and recruitment cohort allocation of individuals^11,49^. Size structure data from Lizard Island revealed that this population represents various size classes (100–600 mm), with no single dominant size class (i.e., recruitment cohort). This suggests that recruitment to this population has occurred over an extended, multi-year period (e.g., Pratchett^11^), rather than through a single mass recruitment pulse (e.g., Zann et al.^67^). This insight into historic recruitment processes can act to establish what putative drivers may have played a role in the initiation of this population irruption. For example, our results do not support the idea that an episodic event (e.g., a flood) caused increased recruitment (Terrestrial Runoff Theory), since there is no dominant peak in recruitment documented in this study, but rather a range of different size classes including recent recruitment^22,24^. On the contrary, our findings suggests that a prolonged accumulation of individuals was/is increasingly adding progeny to the population pool and thereby causing the population size increase. It remains unknown what processes are driving this continually elevated recruitment, although high rates of larval retention due to weak and bidirectional currents in this area may be a contributing factor^30,31,68,69^. Thus, an improved understanding of the larval supply and recruitment dynamics that precede adult population build-up may provide broadly applicable insights into the precise cause(s) of population irruptions.

The data on size structure documented in this study also provide an important insight into the reproductive capacity of the building population, as fecundity of CoTS is strongly size-dependent, whereby reproductive capacity increases exponentially with diameter, particularly in females^14^. Based on the large average size of the population recorded in 2022, which includes high proportions of large-bodied individuals in the 400−600 mm size classes, we can extrapolate that at least half of the female population will be producing upwards of 20 million oocytes per individual, per spawning season^14^. As a result, a substantial number of competent larvae arising from this population will likely settle and recruit into CoTS populations at Lizard Island and well-connected northern GBR reefs every upcoming summer^30,70,71^. While such significant larval supply inherently contributes to the spatial spread of this population irruption, it could also result in a positive feedback loop at Lizard Island, whereby high rates of self-recruitment further reinforce, if not accelerate, localised population growth^29^. These insights on size frequency and reproductive potential have important application for the prioritisation and allocation of population control efforts, because the targeted control of a building population made up of larger individuals (such as Lizard Island), may prove more efficient and effective in slowing the progression of population irruptions than targeting a higher density population which is made up of smaller individuals.

In conclusion, this study has provided strong evidence that the anticipated renewed population irruption of CoTS (e.g., Babcock et al*.*^34^) has already commenced in the northern GBR. The novel monitoring approach developed to reveal this proliferation of CoTS provides an important opportunity for early intervention, and will enable improved understanding of the patterns and processes involved in initiation of population irruptions^47,72^. Considering the spatiotemporal dynamics and severe impact of previous population irruptions^34^, suppression of population growth at key locations such as Lizard Island may contribute to slowing the spread of widespread irruptions^73^. Further in-depth monitoring of CoTS populations in the northern GBR and other regions may provide important insights into the irruptive population dynamics of CoTS, and where, when and why CoTS population irruptions initiate.

## Data Availability

All data analysed as part of this study are available from Research JCU (10.25903/a4k6-yg25).
